# Can the Irisin be a Biomarker for Prostate Cancer? A Case Control Study

**DOI:** 10.31557/APJCP.2020.21.2.505

**Published:** 2020

**Authors:** Rahmi Aslan, Hamit Hakan Alp, Recep Eryılmaz, Zübeyir Huyut, Mehmet Sevim, Şeyhmuz Araz, Kasim Ertas, Kerem Taken

**Affiliations:** 1 *Department of Urology, *; 2 *Department of Medical Biochemistry, Faculty of Medicine, Van Yuzuncu Yıl University, 65080-Van, Turkey. *

**Keywords:** Adipokine, biomarker, irisin, prostate cancer (PCa)

## Abstract

**Aim:**

There is much evidence of an association between cancer and irisin that is an adipokine. This study researched on the relationship between prostate cancer (PCa) and irisin levels, and whether irisin can be used as a biomarker in the diagnosis of PCa.

**Materials and Methods:**

For the study groups, 50 primary PCa patients and 30 healthy male subjects were included in the PCa and healthy control groups, respectively. All volunteers in the healthy control group were screened for prostate cancer and other malignancies and chronic diseases. Volunteers who were determine to be completely healthy were included for healthy control group. In the serum samples of the subjects were measured free PSA, total PSA and irisin levels. Irisin levels were compared separately in terms of the Gleason scores and T stage. In addition to intergroup comparisons, the ROC curve for the irisin was plotted and power analysis was performed.

**Results:**

Free and total PSA levels in the PCa group were significantly higher compared to the healthy control group (p<0.05). In addition, irisin levels in the PCa group were significantly lower than in the healthy control group (p<0.05). There was no significant difference between irisin levels in the groups classified in terms of Gleason scores (p>0.05). When the cut-off value was taken as 8.1, the sensitivity and specificity of irisin for PCa were as 80.5% and 90%, respectively.

**Conclusion:**

The results of this study indicate that the levels of irisin in the PCa group are considerably reduced and irisin may be used as a biomarker as well as free and total PSA.

## Introduction

Irisin is a myokine/adipokine that has been identified in recent years and is widely study worldwide. Irisin is produced by proteolytic cleavage of protein 5 (FNDC5) that is included fibronektin tip III domain (Boström et al., 2012). It is synthesized in many tissues including skeletal muscle and fat cells, and participates in the regulation of lipid and glucose metabolism (Aydin, 2014). Since irisin regulates fat metabolism, it plays an important role in the emergence and development of obesity, insulin resistance associated with obesity, diabetes, non-alcoholic fatty liver disease and other metabolic diseases (Aydin et al., 2016; Zhu et al., 2018). By increasing levels of uncoupling protein-1 (UCP1), Irisin converts white adipose tissue cells into brown adipose tissue cells and reduces obesity (Boström et al., 2012). Many studies in recent years clearly show that there is a connection between irisin and cancer. Cancer increases the energy consumption by increasing the metabolism rate of the organism (Kulluoğlu et al., 2019). Increased amounts of the irisin have been determined in some gastro-intestinal cancers, some benign tumors and endometriosis tissues (Kullooğlu at al., 2016; Aydin, 2016). On the other hand, in breast cancer, a cancer associated with obesity and hormones such as prostate cancer, serum irisin levels were found to be significantly lower than healthy controls (Provatopoulou et al., 2015). In an another study, Irisin has been shown to regulate division and proliferation in prostate cancer cells. (Tekin et al., 2015). 

Factors such as age, family history, smoking, sedentary lifestyle and obesity are blamed in the etiology of prostate cancer (PCa). Obesity in particular is known to be an important risk in both prostate cancer and many other cancers (including colon, ovary, breast, esophagus and pancreas) (Abdulhussein et al., 2018; Bultman, 2018). In the etiology of the PCa, the insulin/insulin-like growth factor-1 (IGF-1) axis, sex hormones and adipokines are also blamed (Roberts et al., 2010; Smith et al., 2018). Adipokines play a role in the progression of different types of cancer by contributing to the onset of inflammation and carcinogen formation. Therefore, adipokine levels may be useful for cancer diagnosis and prognosis. On the other hand, changes in the secretion of adipokines have been reported to be closely associated with prostate cancer (Fryczkowski et al., 2018; Hu et al., 2019).

This study is the first clinical study to investigate the relationship of irisin with prostate cancer (PCa). The aim of the study was to determine whether serum irisin levels are related to PCa and whether it can be used as a biomarker for PCa.

## Materials and Methods

*Study design*

Fifty primary PCa patients (patient group) and 30 healthy male subjects (age matched) were included in this prospective case control study. Patients were selected from the urology clinic of a tertiary university hospital. Healthy men included in the control group were selected from hospital staff and patient relatives. Written informed consent was obtained from patients and volunteers. Ethics committee approval (ethics committee approval number: 1912201814) was obtained from Van Yüzüncü Yıl University Medical Faculty Non-Drug Clinical Practices Local Ethics Committee for this study. All volunteers in the control group were screened for prostate cancer and other malignancies and chronic diseases. Volunteers who were determine to be healthy were included in the study. Patients with comorbidities such as cardiovascular diseases, metabolic diseases, diabetes, chronic kidney disease, other malignancies, acute and chronic infective disease, chronic obstructive pulmonary disease, other ischemic and immunosuppressive diseases were excluded. In addition, those who performed routine heavy exercise were excluded from the study.

Age, prostate volume, international prostate symptom score (IPSS), body mass index (BMI) and histopathological data were recorded for both groups. 

*Sample collection and analysis*

Blood samples were obtained from peripheral venous vessels of the PCa and healthy control groups. Blood samples were collected in yellow-cap biochemistry tubes. Blood samples were centrifuged at 3,000 rpm for 10 minutes. Serum samples obtained were kept at -80°C until biochemical analysis. Serum irisin levels were mesured in accordance with the kit prospectus by commercially purchased ELISA (enzyme-linked immunosorbent assay) kits (YL biont biotech Co. Shanghai) in PCa and healthy control groups. Serum cancer markers total PSA and free PSA levels were also measured by chemiluminescence microparticle immunological assay on routine biochemistry analyzer (Abott Architect İ4000 SR). 

*Statistical analysis*

IBM SPSS 20 program was used for statistical evaluation. The normal distribution of the data was checked by Shapirow Wilcs test and equal variance (F-test). Data were expressed as mean and standard deviation (mean ± SD). The independent sample T test was used to determine whether the difference between PCa and healthy control group was significant. We used Spearmen Correlation test for detected correlation between irisin and prostate volume. The statistical significance was considered for p<0.05. In addition, the recipiant operator curve (ROC) analysis was performed. Furthermore, power analysis was performed with G power computer program. The effect size was 1.56 and the strength of the study was found to be 96% for 50 patients of PCa and 30 volunteer of the healthy control. 

**Table 1:** Descriptive Data of the Prostate Cancer and Healthy Control Group

	Prostate Ca N=50 (Mean±SD)	Healthy control N=30 (Mean±SD)
Age (year)	65.5±7.35	65.0±10.8
BMI (kg/cm^2^)	27.5±3.59	26.9±2.51
PV (mL)	71.4±33.3	23.1±8.77*
IPSS	17.1±5.51	0.66±1.32*

PV, Prostate volume; IPSS, International prostate symptom score; BMI, Body Mass Index; *, Statically different than the prostate Ca group (p<0.001)

**Table 2:** The Levels of Irisin, Total PSA and Free PSA in the Groups

	Prostate Ca N=50 (Median±SEM) (min-max)	Healthy control N=30 (Median±SEM) (min-max)
Irisin (pg/mL)	6.92±2,44 (1.47-11.2)*	13.5±6.21(6.58-30.2)
TPSA (ng/dL)	39.6±26.95 (4.00-125)*	1.77±0.81 (0.10-2.5)
FPSA (ng/mL)	1.71±1.76 (0.01-39.0)*	0.06±0.04 (0.01-0.80)

*p(<0.05): It is significant according to the healthy control group

**Figure 1:**
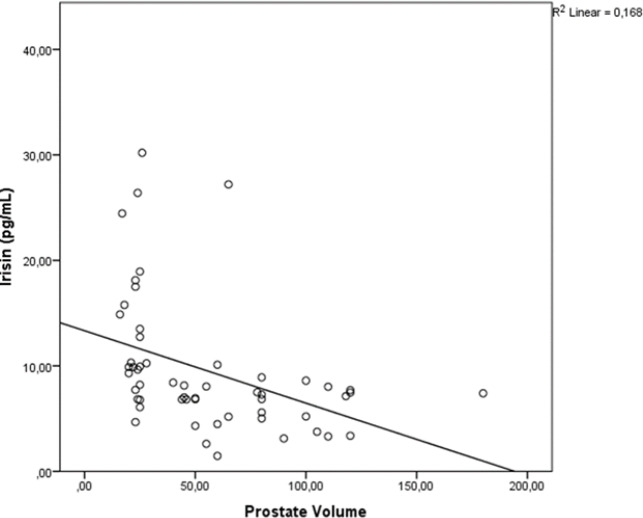
The Negative Correlation between Irisin and Prostate Volume: (Scotter/Dot graphy)

**Table 3:** The mean ± sd of Irisin Level for Different Categories of Gleason and T Stage Score

Gleason score	<7 (n=15)	=7 (n=7)	>7 (n=11)	
Irisin (pg/mL)	5.84±2.16	5.91±2.67	6.71±1.33	
T stage	T1 (n=12)	T2 (n=8)	T3 I(n=5)	T4 (n=5)
Irisin (pg/mL)	5.18±1.72	6.72±2.57	6.24±1.65	7.01±1.11

**Figure 2:**
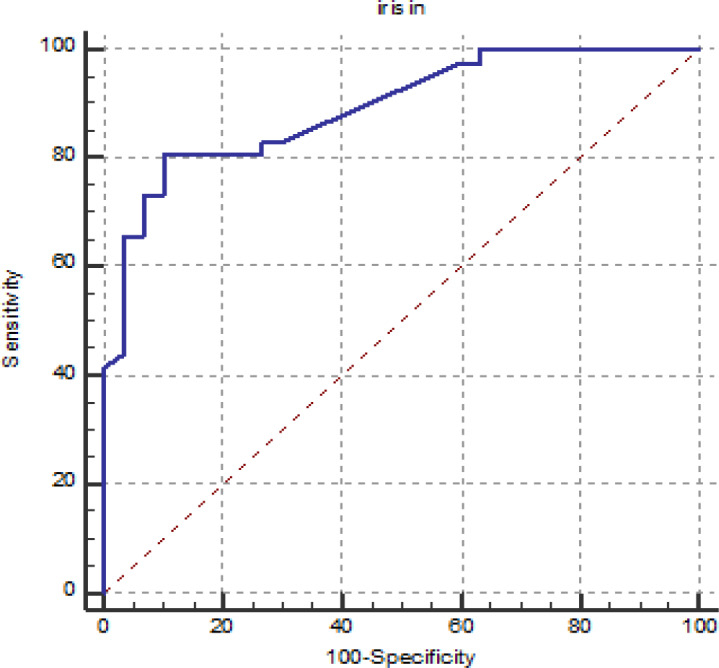
The Patient/non-Patient Discrimination of the Iris Levels: Recipiant Operator Curve (ROC)

## Results

*Descriptive data*

The basic demographic and clinical characteristics of PCa and healthy controls groups are shown in Table 1. Mean age and BMI values were similar in both groups. The mean prostate volume and IPSS values were lower in the healthy control group compared to the PCa group (p<0.05).

*Comparison of mean Irisin and PSA values in PCa and control groups*

The mean irisin level in the PCa group was significantly lower than in the healthy control group (6.92±2.44 and 13.5±6.21, respectively, (p<0.05). Total and Free PSA levels in the PCa group were significantly higher than in the control group (Table 2). There was a negative relationship between the irisin levels and PSA values in the CaP group compared to the healthy control group (p <0.05). 

*The relationship between iris levels and tumor characteristics*

The relationship between serum irisin levels and tumor characteristics was evaluated in the PCa group (Table 3). We did not find any relationship between irisin levels and Gleason scores, which is the most important histopathological parameter showing PCa aggression. Also, there was no significant relationship between the stage of cancer and irisin levels. 

*Relationship between prostate volume and irisin, and ROC analysis*

In addition, we investigated correlation between the irisin and prostate volume levels. There was a significant negative correlation between the irisin and prostate volume (r=0.538 and p<0.001, (Figure 1). 

The patient/non-patient discrimination of the iris levels was performed by Recipiant Operator Curve (ROC) analysis (Figure 3). According to ROC analysis, the area under the curve (AUC) was found 0.892. When the cut-off value was taken as 8.91 pg/mL, the sensitivity and specificity were found to be 80.5% and 90.0%, respectively (Figure 2). 

## Discussion

Nowadays, PCa is considered to be one of the most important health problems of the male population. PCa is the second most frequently diagnosed cancer worldwide with more than 1.1 million new cancer cases each year. It accounts for approximately 15% of all diagnosed cancers (Ferlay et al., 2012). In addition, PCa is the second most common cause of cancer deaths in men (Heidenreich et al., 2011). Testosterone and some other hormones are known to play a role in the etiology of prostate cancer. On the other hand, many author have reported that obesity is an important risk factor for cancer development. Therefore, some hormones that reduce the harmful effects of obesity have been studied quite a lot (Moller et al., 2015; O’Rurke et al., 2014).

Many studies have been investigated the relationship of FNDC5 / irisin with obesity, chronic kidney disease, Type 2 Diabetes Mellitus, and chronic diseases (Zhang et al., 2013; Wen et al., 2013; Liu et al., 2013). Recently, many studies have been also conducted on the relationship between Irisin and cancer. Us Altay et al., (2016) in their experimental study showed that serum iris levels were significantly increased in rats with gastric cancer. In a study investigating the relationship between gastrointestinal (GIS) cancers and irisin; Aydın et al., (2016) detected significantly increased irisin levels in GIS cancer tissues except for liver cancer. Provatopoulou et al., (2015) reported that serum iris levels in breast cancer patients were significantly lower than in healthy controls. The authors found a significant relationship between iris and breast cancer according to multivariate and univariate analyzes. In a study of breast cancer cell culture, treatment with irisin was reported to cause a decrease in the amount of human malignant aggressive breast epithelial cells (Gannon et al., 2015). In a study on osteosarcoma cell culture, Gang Kong et al., (2017) reported that iris inhibits proliferation, invasion and migration of osteosarcoma cells.

Although irisin has been investigated in relation to many benign and malignant neoplasms, we have not found any studies investigating its relationship with prosat cancer. This study is a -first in this regard. In this prospective controlled study, we measured serum iris levels of prostate cancer and healthy volunteers. Serum irisin levels in patients with PCa were significantly lower than healthy volunteers. In a previous study, irisin levels were found to be associated with breast cancer stage and tumor volume while it was not relation with tumor markers such as CEA, CA15-3 (8). Moon et al., (2014) reported that irisin has no significant effect on the malignancy potential and cell proliferation of colon and endometrial cancer cells. In another similar study, Shi et al., (2017) reported that irisin significantly increased cell proliferation, invasion and migration in liver cancer. They also showed that irisin decreased the sensitivity of doxorubicin to liver cancer cells. In contrast to the above study, Shao et al., (2017) showed that irisin inhibited proliferation, migration and invasion of lung cancer cells. On the other hand, another study showed that the irisin suppresses the growth of pancreatic cancer cells (Liu et al., 2018). All these studies show that iris has positive or negative effects on human cancers. However, our knowledge in this subject is still very limited and contradictory. When the data obtained were evaluated in total, this evaluations indicate that malignancies related to hormone levels such as breast and prostate cancer may be associated with decreased irisin levels. In our study, levels of low irisin in PCa patients compared to healthy controls indicate that it is a result of malignancy and that high irisin levels may be suppress PCa disease. 

In this study we did not found a significant relationship between irisin levels and gleason score and cancer T stage. According to current European guidelines, there is a variety of evidence-based treatment options for PCa patients and reliable biomarkers are urgently needed to guide the pre-treatment decision process for this growing patient subgroup (Mottet et al., 2017). In our study, according to the ROC analysis, the sensitivity and specificity of irisin for the diagnosis of PCa were found to be 80.5% and 90.0%, respectively, it indicates that this test with larger series of studies may be used as an adjunct biomarker for the diagnosis of PCa. 

In conclusion, our data show that serum irisin levels are significantly lower in patients with PCa. However, it also shows that there is no relationship between irisin and cancer stage and Gleason scores.

Although this study is a prospective controlled study, it has some limitations. The small number of patients and single center are important limitations. However, according to our knowledge, this is the first study to investigate the relationship between PCa and irisin. In order to clarify the details of the relationship between iris and prostate cancer, there is needed researches with more multicentre and a higher number of subjects. In addition, does PCa result from low levels of irisin or is it decreasing as a result of malignancy? This issue needs to be elucidated with more detailed studies. 
